# Myocardial perfusion imaging with retrospective gating and integrated correction of attenuation, scatter, respiration, motion, and arrhythmia

**DOI:** 10.1007/s12350-023-03374-5

**Published:** 2023-09-27

**Authors:** Kenichi Nakajima, Takayuki Shibutani, Francesc Massanes, Takeshi Shimizu, Shohei Yoshida, Masahisa Onoguchi, Seigo Kinuya, A. Hans Vija

**Affiliations:** 1 Functional Imaging and Artificial Intelligence, Kanazawa University, Kanazawa, 920-8640 Japan; 2 Quantum Medical Technology, Institute of Medical, Pharmaceutical and Health Sciences, Kanazawa University, Kanazawa, Japan; 3 Siemens Medical Solutions USA, Inc. Molecular Imaging, Hoffman Estates, IL USA; 4 Department of Cardiovascular Medicine, Kanazawa University Graduate School of Medical Science, Kanazawa, Japan; 5 Department of Nuclear Medicine, Kanazawa University, Kanazawa, Japan

**Keywords:** Quantitative myocardial perfusion imaging, coronary artery disease, left ventricular function, data driven gating, respiratory motion correction, list-mode data acquisition

## Abstract

**Background:**

Absolute quantitative myocardial perfusion SPECT requires addressing of aleatory and epistemic uncertainties in conjunction with providing image quality sufficient for lesion detection and characterization. Iterative reconstruction methods enable the mitigation of the root causes of image degradation. This study aimed to determine the feasibility of a new SPECT/CT method with integrated corrections attempting to enable absolute quantitative cardiac imaging (xSPECT Cardiac; xSC).

**Methods:**

We compared images of prototype xSC and conventional SPECT (Flash3D^TM^) acquired at rest from 56 patients aged 71 ± 12 y with suspected coronary heart disease. The xSC prototype comprised list-mode acquisitions with continuous rotation and subsequent iterative reconstructions with retrospective electrocardiography (ECG) gating. Besides accurate image formation modeling, patient-specific CT-based attenuation and energy window-based scatter correction, additionally we applied mitigation for patient and organ motion between views (*inter-view*), and within views (*intra-view)* for both the gated and ungated reconstruction. We then assessed image quality, semiquantitative regional values, and left ventricular function in the images.

**Results:**

The quality of all xSC images was acceptable for clinical purposes. A polar map showed more uniform distribution for xSC compared with Flash3D, while lower apical count and higher defect contrast of myocardial infarction (p = 0.0004) were observed on xSC images. Wall motion, 16-gate volume curve, and ejection fraction were at least acceptable, with indication of improvements. The clinical prospectively gated method rejected beats ≥20% in 6 patients, whereas retrospective gating used an average of 98% beats, excluding 2% of beats. We used the list-mode data to create a product equivalent prospectively gated dataset. The dataset showed that the xSC method generated 18% higher count data and images with less noise, with comparable functional variables of volume and LVEF (p = ns).

**Conclusions:**

Quantitative myocardial perfusion imaging with the list-mode-based prototype xSPECT Cardiac is feasible, resulting in images of at least acceptable image quality.

**Supplementary Information:**

The online version contains supplementary material available at 10.1007/s12350-023-03374-5.

Single-photon emission-computed tomography (SPECT) imaging has been a standard method since the 1980s and electrocardiographic (ECG) gating in the 1990s added the ability to simultaneously evaluate myocardial perfusion and function. Since then, several limitations of SPECT imaging have been revealed in terms of quantitation and repeatability. Currently, the most prevalent means of evaluating SPECT images remain with visual interpretation, semiquantitative scoring, and left ventricular function assessment.^1–3^ Such visual and quantitative assessments have been routinely adopted in multicenter studies and clinical guidelines using conventional imaging and software-based methods.^4,5^

Image reconstruction with filtered back projection (FBP) is used in 49% of Japanese institutions, ordered-subset expectation maximization (OSEM) in 30% , and both FBP and OSEM in 21%.^6^ Furthermore, both attenuation (AC) and scatter (SC) corrections are routinely used in 31% of institutions with an iterative reconstruction method. SPECT reconstructions done by FBP are not corrected for attenuation and scatter. Nuclear cardiologists require training to adequately interpret myocardial perfusion images, while considering the possibility of image artifacts. In the process of iterative image reconstruction, we need to consider the impact of inadequate compensation of patient-induced tomographic inconsistencies. A tomographic inconsistency occurs when the data model does not reflect the reality of the actual acquisition and can be caused by, e.g., patient attenuation, scatter, beating hearts including arrhythmia, respiratory, and body movements. Tomographic inconsistencies might develop image artifacts, becoming typically more noticeable as iteration updates increase. Although some of the image degrading factors have been investigated individually, to our knowledge, there has not been an evaluation of the impact on image quality in myocardial SPECT when all these corrections are used simultaneously. This is of importance, as the statistical quality of data can only justify a limited number and degree of corrections. Each correction method requires estimable parameters, hence, the primary concern that corrections follow the “do-no-harm” paradigm, i.e., guarding against insufficient data with too many parameters that might yield to instability, revealing as artifacts and poor reproducibility and repeatability.

The present study aimed to determine the feasibility of integrated and automated corrections attempting to enable absolute quantitative cardiac imaging, improve reproducibility and repeatability, and to find ways to further improve SPECT image quality by mitigating patient attenuation, scattering, overall tomographic inconsistency due to patient body motion and respiratory motion. We used retrospective gating to more effectively account for cardiac contraction with/without arrhythmia, and allowed for continuous rotation, thus, maximizing the data collection.

## Methods

### Patients

This study included 56 patients (age, 71 ± 11 years; male, 79%) with definite or suspected CAD who were assessed by myocardial perfusion imaging (MPI) (Table 1). Old myocardial infarction was clinically diagnosed in 24 (44%) patients (body mass index [BMI], 23 ± 5 kg/m^2^) who were included for comparisons of perfusion at rest. The summed rest score (SRS) determined automatically using standard databases by the Japanese Society of Nuclear Medicine normal database was 6.9 ± 6.6 (range 0-24).^7^

**Table 1:** Demographics of patients

	n (%), or means ± standard deviation
Number (n)	56
Age (y)	72 ± 11
Male sex (n (%))	44 (79%)
Height (cm)	(M) 166 ± 6, (F) 152 ± 7
Weight (kg)	(M) 65 ± 14, (F) 48 ± 12
Body mass index (kg/m^2^)	(M) 24 ± 4, (F) 21 ± 6
Stress type (N)	Adenosine 55, rest only 1
Radiopharmaceuticals	^99m^Tc-MIBI 49, ^99m^Tc-tetrofosmin 7
*Defect scores*	
Summed stress score	9.9 ± 6.7
Summed rest score	7.2 ± 6.5
Summed difference score	3.1 ± 2.6
*Diagnosis*	
Coronary artery disease	45 (80%)
Heart failure	2 (4%)
Pre-operative evaluation	2 (4%)
Non-ischemic metabolic disease (Chronic kidney disease, diabetes mellitus)	7 (13%)
Myocardial infarction	24 (44%)

### Imaging protocol

The resting part of stress-rest MPI was used from a one-day protocol study. All patients fasted during the morning and refrained from taking any medications that might affect the MPI results. The patients were then assessed under stress by adenosine SPECT imaging using 260 MBq (7 mCi) of ^99m^Tc-methoxy-isobutyl-isonitirile (MIBI) (n = 49) or tetrofosmin (n = 7), and at rest with 700 MBq (18 mCi) of the same tracers.

### Acquisition of SPECT data

Conventional SPECT images were acquired at Kanazawa University Hospital using a Symbia Intevo 16 SPECT/CT and our routine clinical protocol comprising of AutoForm™ low-energy high resolution collimators with a 15% peak window, centered at 140.5 keV for ^99m^Tc and a 15% lower abutting scatter window (standard dual-energy window method). Sixty projection views over 360° with 6° step-and-shoot sampling were accumulated using a 64 × 64-matrix with a zoom factor of 1.45 (6.6-mm pixels), 35 seconds per view in a tight circular orbit (rotation radius of 24 cm).

A 3%-NIST traceable calibrated ^57^Co-source was used to calibrate the SPECT imager per xSPECT Quant ^TM^ protocol. The prototype 1 xSPECT Cardiac (xSC) acquired in continuous rotation and list-mode with 120 projection views over 360° with 3° sampling in a 256 × 256 matrix (2.4-mm pixels), 15 seconds per view, non-circular orbit. Thus, the double-scan procedure consisted of current clinical and research protocols. The sequence of scan was conventional resting SPECT followed by xSC data acquisition. The R-wave served as a trigger for conventional prospective electrocardiogram (ECG) gating with a fixed width beat window (20%) and enabled auto-tracking of RR intervals that are divided into 16 gated frames. Nuclear medicine technologists appropriately set a wider acceptance window for patients with arrhythmia such as frequent premature beats and atrial fibrillation. In the xSC list-mode acquisition, the ECG measured physiological trigger events allowed gated continuous rotation followed by retrospective gating during reconstruction.

### SPECT image reconstruction

We used our clinical standard Flash3D^TM^ for conventional ungated and gated reconstructions, with a distance-dependent, 3D-Gaussian collimator model for the point spread function within OSEM at 120 updates (12 subsets and 10 iterations) based on corrected framed data. A 3D-Gaussian filter at a full-width at half maximum (FWHM) of 13.2 mm was used for post smoothing. We do not typically use CT attenuation correction in clinical practice at our hospital.

The xSC reconstruction was based on xSPECT Quant^TM^ from list-mode data using a measured 3D point response function, 3D rotation with adaptive class-standard gantry deflection, CT-derived attenuation correction (AC), and energy window-based scatter correction (SC) as described by Vija *et al*..^8–10^ In this work, we chose the OSEM-based xSPECT Quant reconstruction, as it yields a more similar resolution and noise impression to the Flash3D reconstruction.

The xSC reconstruction contained retrospective gating, *inter-view*^2^ and *intra-view*^3^ motion correction, which are detailed below.

*Retrospective gating:* Retrospective gating enabled efficiency improvement by allowing continuous rotation in gated studies thereby maximizing the counts utilized for each view. In the xSC reconstruction, a beat histogram was curated using automated data analysis for outliers,^11^ where the acceptance window was defined with ± 3 root-mean square of the median in an iterative process run to convergence (“curation”). The curation rejected so defined premature ventricular contraction and 1 beat after it. This process was an attempt to maintain the maximum number of counts possible even when abnormal cardiac rhythms presented a challenge to the prospective approach. In addition, it reduces tomographically inconsistent assignments of gate to angle that may occur in the prospective scheme resulting in, e.g., radial blurring and then potentially impacting LVEF assessment.^12^ Albeit any number gates are in principle possible, we also selected 16 gated frames as a good trade-off between volume curve sampling and statistical power. Each xSC gate was reconstructed with attenuation and scatter correction.

We checked for beat histogram consistency in the back-to-back clinical and dedicated-research acquisitions, to exclude a temporal bias due to sequenced acquisition. We applied the same prospective gating and framing algorithm to the list-mode data as implemented in the clinical product which framed them with a 20% acceptance window and auto-tracking. We examined six patients with frequent arrhythmia and outlier beat lengths, and 12 without arrhythmia during data acquisition.

*Inter-view* motion “correction”: Its goal was to reduce tomographic inconsistencies, mitigating a build-up of image artifacts as updates progress. It was designed to be automated and compatible with iterative reconstruction. However, it is limited by poor count statistics and the fundamental limitations of the image formation. The key difference between conventional and this new approach is briefly described in the following (see details in supplement file Section A). The current commercially available proprietary semi-automatic method was based on estimation of axial and transaxial motion in each projection view to mitigate cardiac motion induced by patient motion or cardiac creep. Transaxial and vertical motion estimations were based on sinograms and linograms, respectively, and the projections were shifted view-by-view in axial and transaxial direction prior to reconstruction.^13,14^ Fundamentally, the method suffered when significant change of contrast of the myocardial projection occurred due to patient-specific factors. The contrast can fall so low that edge detection and subsequent fits become unreliable in some views, and potentially inducing erroneous shifts in these views. Manual intervention is often needed, which is subjective and rarely repeatable, resulting in a trial-and-error reconstruction approach. The *inter-view* motion correction overcomes this issue using a fully automated method applicable to all study types including cardiac studies, which allows for rigid axial and transaxial shifts measured within and was designed for the iterative reconstruction process.^15^ The shift vectors were estimated in a dedicated automated reconstruction preceding the user-adjustable target reconstruction. This dedicated reconstruction named “Improving Tomographic Consistency” (ITC)-reconstruction used the same update method, data model, and enables AC and SC as the target xSC reconstruction. It was not user adjustable and solely for the purpose of shift vector estimation. The xSC reconstruction restarted using the shift vectors from the preceding ITC reconstruction by updating the 3D rotation matrix, thereby improving tomographic data model consistency for all views. Strictly speaking this approach represents a motion correction only if the tomographic inconsistency was caused by a rigid-body axial and transaxial motion, otherwise it is merely a mitigation.

*Intra-view* motion “correction”*:* This refers to mitigating for motion effects during the acquisition of a view for the duration of the dwell time at the specific viewing angle (see details in supplement file, Section B). The method can mitigate the impact of motion during the dwell time for both continuous and discontinuous rotation if the statistical data quality allows it. Respiration is typically the main cause for such motion. However, respiratory-induced cardiac motion may also show up as view-to-view jitter, a type of inconsistency appearing over neighboring views. This type of residual *inter-view* jitter is more noticeable when the dwell time is on the order of the respiration period, which can be addressed by the subsequent use of *inter-view* motion correction. We used the non-linear Laplacian Eigenmap data-driven dimensionality reduction approach based on the assumption that the respiration information data laid on a lower-dimensional manifold.^16–18^ The data-driven gating subdivided the projection view into respiratory-gated views (RGV). In essence, the dwell time was split into six estimated respiratory gates of unequal temporal duration to create six RGV’s, enabling an estimate of the center-of-light 4 (COL) motion of the field of view (FOV) in each RGV, which were then shifted to a common reference location within the FOV, prior to summation of all RGV’s into the projection frame that was subsequently used in the reconstruction. This approach attempted to mitigate respiration-induced blurring and shape deformation, and was limited by poor count statistics and, thus, mandatory limitations of the number of degrees of freedom in the underlying model. Thus, the method only allowed for axial rigid translation.

### Effect of motions on cardiac phantom

We assessed the potential improvement on image quality of the *inter* and *intra-view* motion correction method by applying patient-derived shift vectors to a perfectly still phantom dataset, thereby inducing the artifacts expected of the respective type of inconsistency. We used data from an anthropomorphic normal male cardiac phantom (Data Spectrum Corporation, Durham, NC, USA) acquired in the same configuration as the xSC. The shift vectors were extracted from patients, and histograms of all the transverse (x) and axial (y) shift vectors of the patient data were generated.

### Visual analysis of image quality

Two experienced nuclear medicine physicians and a technologist visually assessed vertical and horizontal long- and short-axis images reconstructed by conventional and xSC methods. They compared the quality of blinded images, noise, artifacts, the possibility of false defects and a previous myocardial infarction if it had been clinically diagnosed prior to this study.

### Quantitative analysis

All beat lengths during SPECT data acquisition were analyzed using RR interval histograms, and fractions of beats within the 20%-acceptance window beat length were measured. Images were reconstructed and displayed as long- and short-axis images using 4DM software (INVIA, Ann Arbor, MI, USA) with standard 17-segment polar map display and segmental count (%) calculations. Left ventricular (LV) end-diastolic volume (EDV), end-systolic volume (ESV), and ejection fraction (EF) were compared between prospective and retrospective gating methods. Echocardiographic measurements of volume and EF (modified Simpson method with four- and two-chamber views) within 3 months of an MPI study served as the reference. Cross-sectional and circumferential profiles were analyzed at the mid-portion of transaxial SPECT images as well as slices with perfusion defects to evaluate contrast.

### Ethics Committee approval

The Ethics Committee of Kanazawa University approved this collaborative study. All included patients provided written informed consent to participate.

### Statistics

All data are expressed as means ± standard deviation (SD), and means were compared using the analysis of variance. Categorical variables were analyzed using contingency tables. Segmental counts on polar maps were assessed using paired-comparison tests. Linear regression was calculated using the least-squares method. All data were statistically analyzed using JMP Pro v. 16.0 (SAS Institute Inc., Cary, NC, USA). Values with P < 0.05 were considered significant.

## Results

### SPECT diagnosis

Diagnoses were classified according to findings of perfusion defects and function as large infarction (n = 12, 21%), small to intermediate infarction (n = 12, 21%), heart failure without perfusion defects (n = 8, 14%), and near-normal perfusion and function (n = 24, 43%).

### Image quality

**Figure 1:**
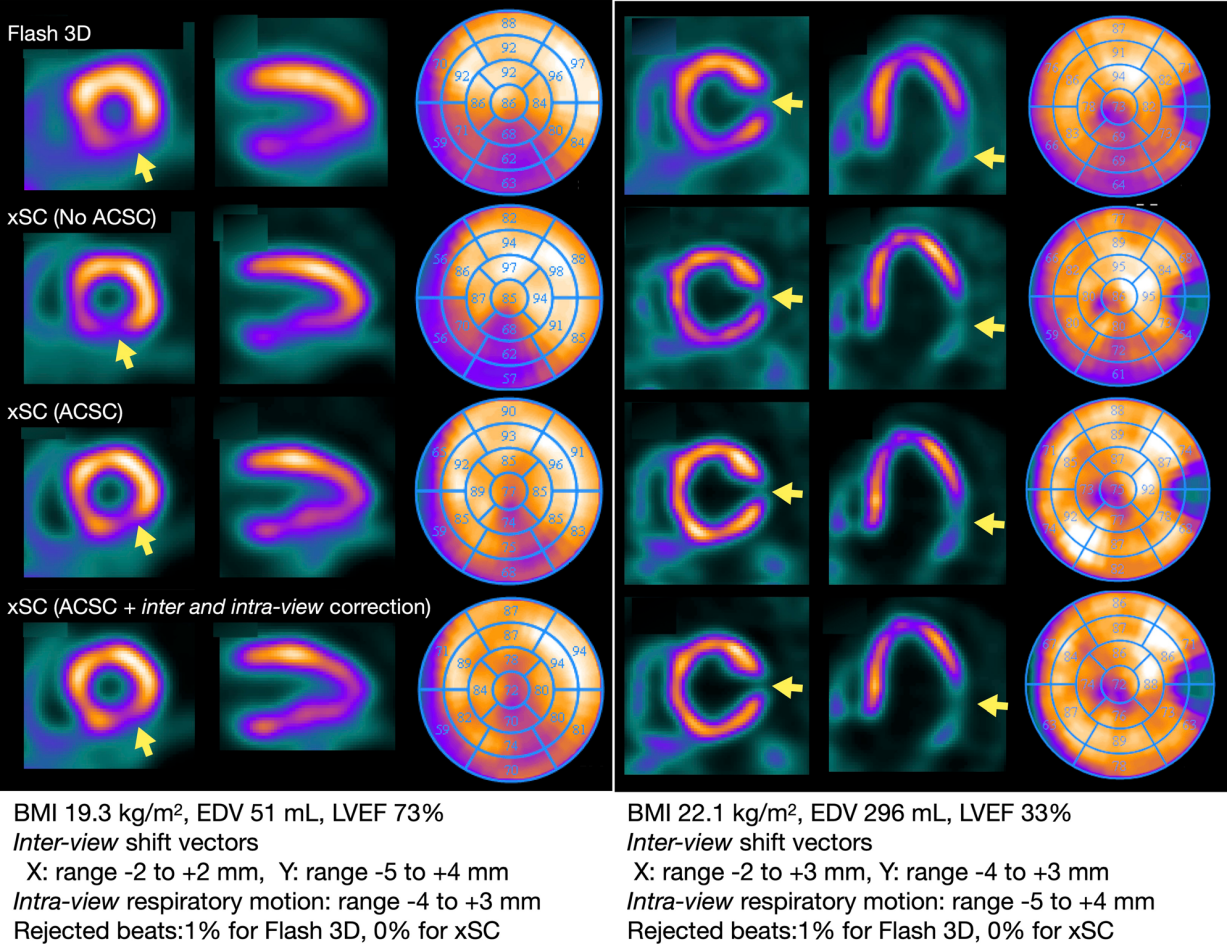
Myocardial perfusion images of two patients processed using OSEM reconstruction without attenuation and scatter corrections and integrated corrections. Reconstruction conditions were OSEM subset 1, iteration 24, and FWHM of Gaussian filter 10 mm. (A) Small heart with inferior old myocardial infarction. (B) Lateral infarction with left ventricular dilation. Body mass index (BMI), left ventricular end-diastolic volume (EDV) and ejection fraction (EF) are shown. Pixels shifts for corrections of projection images to x- and y-directions and respiratory motion are shown. AC, attenuation correction; F3D, Flash3D; ITC, improved tomographic consistency; RC, respiratory motion correction; SC, scatter correction; xSC, xSPECT Cardiac

The visual quality of xSC images was judged as better (44%) and comparable (56%) to those of conventional SPECT images. False defects were not evident in 75% of patients’ images when reconstructed by both methods. However, the conventional method gave a false impression of hypoperfusion in the inferior wall in 23% of the patients. Myocardial infarction was diagnosed by both methods in 20 (91%) of 22 patients. Seven percent of images reconstructed with xSC were considered noisy. In general, xSC images showed thinner myocardial walls, more homogeneous distribution in walls, and higher image contrast than conventional Flash3D images (Figure 1).

The comparison of four reconstruction methods using conventional Flash3D without AC and SC, xSC without AC and SC, xSC with AC and SC, and xSC with all AC, SC, *intra-view* and *inter-view* motion correction is shown in Figure 1. Reduced count of the inferior walls was improved with CT-based AC and SC, and a small infarct was visualized (Figure 1A). Appearance of inferior hot area in xSC with AC and SC was decreased after enabling *inter-* and *intra-view* corrections (Figure 1B).

### Quantitation with polar map

**Figure 2:**
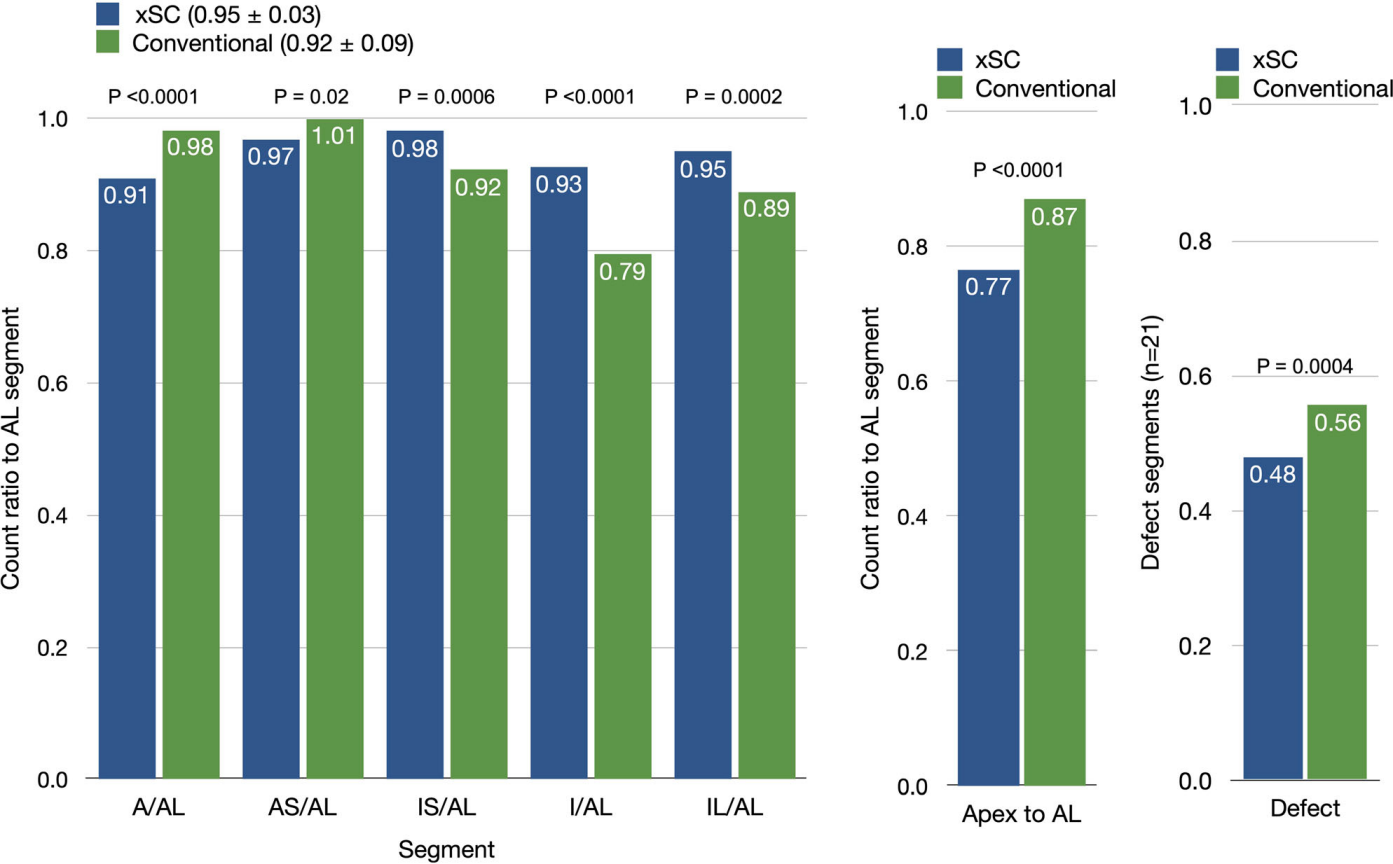
Comparison of segmental counts to anterolateral (AL) count ratio between xSC and conventional methods. (A) Ratio of segmental wall counts to AL in mid segments, (B) Ratio of apical to AL count ratio, (C) Defect segment count to maximum segmental count. P values are calculated by paired t test. Abbreviations of the segments: A, anterior; AS, anterolateral; IS, inferoseptal; I, inferior; IL, inferolateral; AL, anterolateral

Regional counts in walls (except for defect segments) were measured using a polar map (Figure 2). In this analysis, 60 defective segments out of 392 segments were excluded. The defect count was measured at the center of the segments in 21 patients. A comparison of segments to the anterolateral segments revealed that segmental distribution was 0.95 ± 0.03 for xSC and 0.92 ± 0.09 for conventional images. The count ratio of apical-to-anterolateral segment was lower in xSC method than conventional method (p <0.0001). Patients with myocardial perfusion defects had segmental defects of 0.48 and 0.56 in xSC and conventional images (p = 0.0004), respectively. This indicated higher defect contrast in xSC images.

### Circumferential and cross-sectional profiles

**Figure 3:**
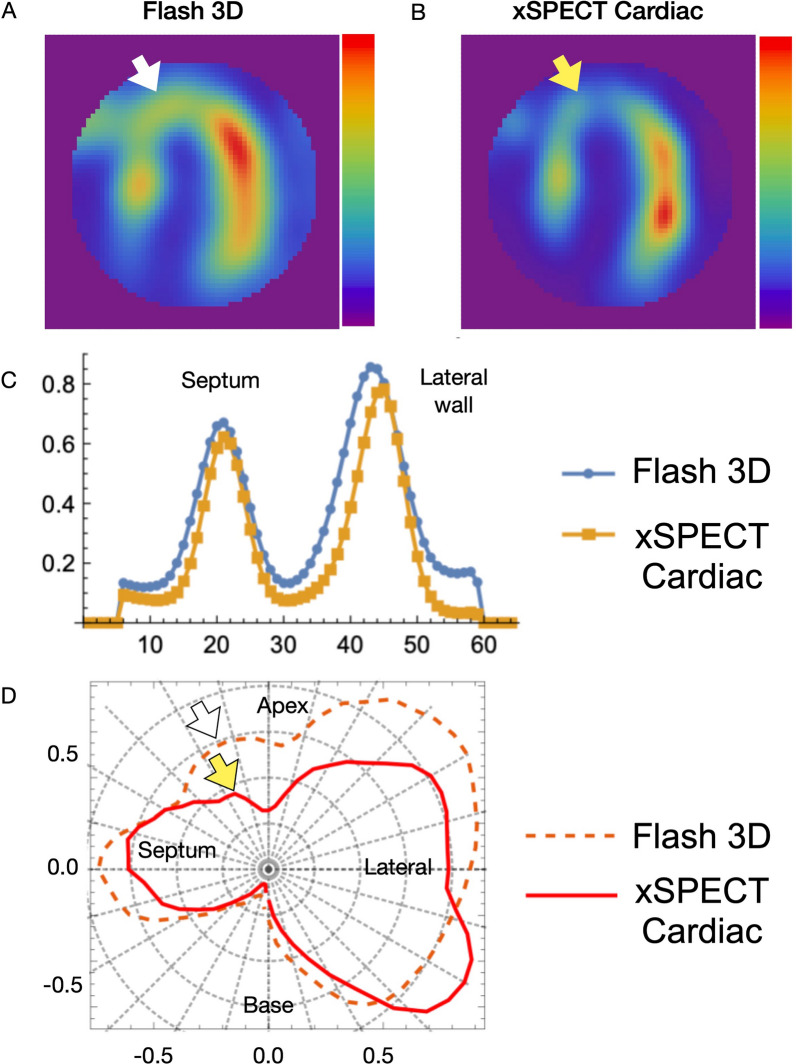
Comparison of SPECT slices in images of patient with anteroseptal infarction. (A) Flash3D and (B) xSPECT. Horizontal cross-sectional and circumferential profile curves (C) shown as polar coordinates (D). Wall thickness, perfusion defect contrast, and LV cavitary counts clearly differ between methods. White and yellow arrows indicate infarction and corresponding profiles on polar plots

Differences in image characteristics can also be determined by profile analysis. Figure 3 shows a patient with anteroseptal myocardial infarction (defect size, intermediate). Profiles and lower defect counts revealed that the infarcted myocardium was thinner in both images. Ratios of the left ventricular cavitary counts to the maximum myocardial count ratio were respectively 14% and 8% using conventional and xSC reconstruction. Average counts in the LV cavity and associated standard deviation were lower in xSC than conventional SPECT images (10.0% ± 6.2% vs. 16.4% ± 8.3%, p < 0.0001).

### Effects of inter-view and intra-view motion corrections

**Figure 4:**
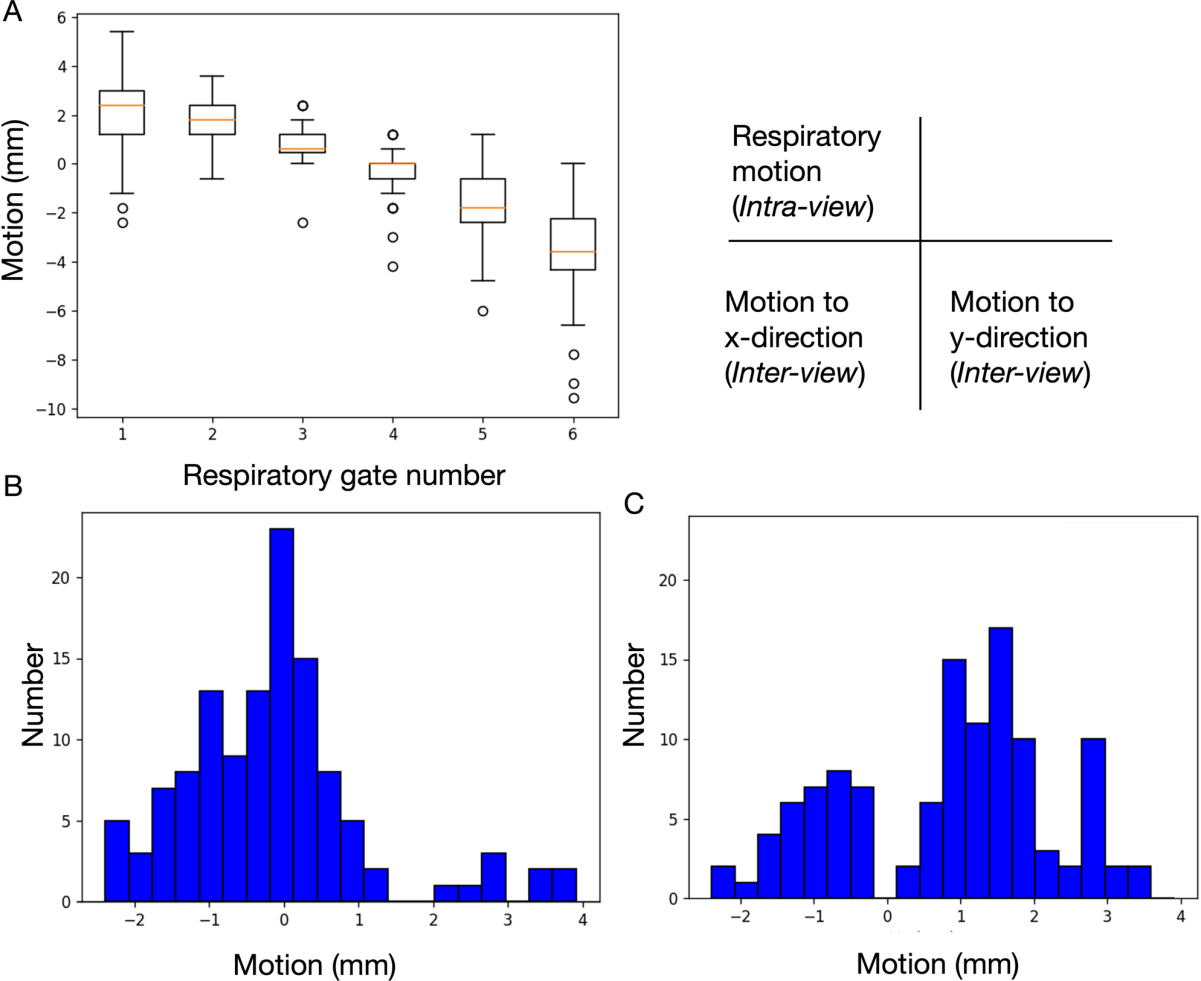
Degree of respiratory motion in each respiratory gate and *inter-view* motion to x- and y-directions in a patient. When all the list-mode data are analyzed, variations of respiratory movements (A) and movements to x- and y-directions (histograms of panels B and C) are notable. Box plots denote median and upper and lower quartiles with outlier datapoints

**Figure 5:**
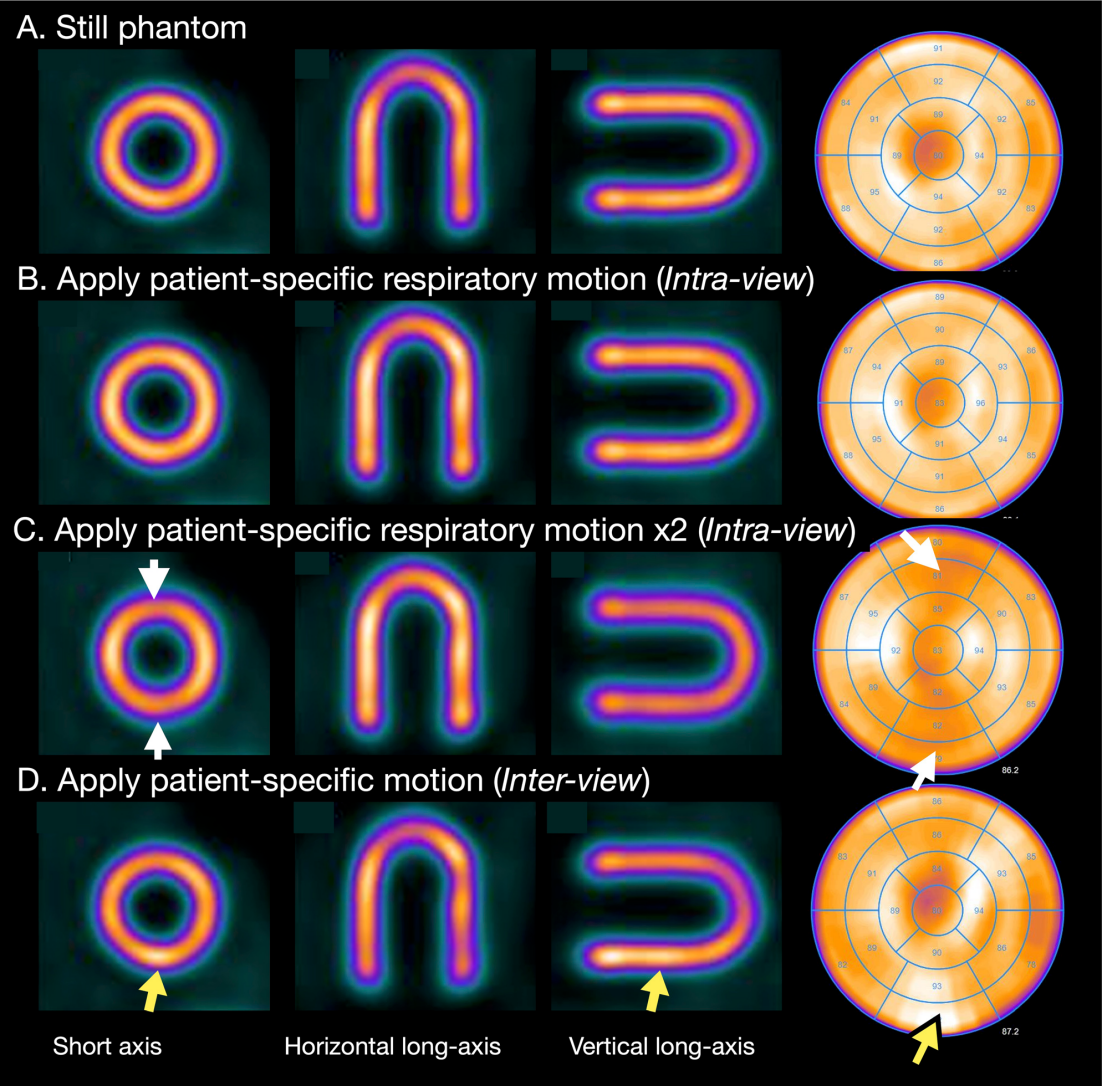
Reconstructed tomographic slices using shift vectors applied to a still phantom (A). The patient-specific respiratory motion shown in Figure 4 was applied to the still phantom (B). When twofold movements were applied during reconstruction, decreased counts in the anterior and inferior region (white arrows) are recognized (C). The patient-specific *inter-view* motion to x- and y-directions also resulted in artifactual heterogeneity and an inferior hot area (yellow arrows, D)

Impact of *intra* and *inter-view* motion correction approach is shown in Figures 4 and 5 as a typical example. The motion vectors were derived from a 77-year-old male patient with old myocardial infarction. When a histogram of all the transverse (x) and axial (y) shift vectors of the patient data in the study were examined, the shifts were found to be within -2 mm and 4 mm (Figure 4). We found that respiratory motion of this range had little impact except for slight blurring as compared with original still phantom image (Figure 5A, B). However, when respiratory motion by twofold (-4 mm to 8 mm) was applied, heterogeneity of distribution was seen (Figure 5C). On the other hand, the *inter-view* motion to x- and y-directions in this case apparently resulted in an artifactual heterogeneity in the polar map distribution (Figure 5 D).

### Left ventricular function

**Figure 6:**
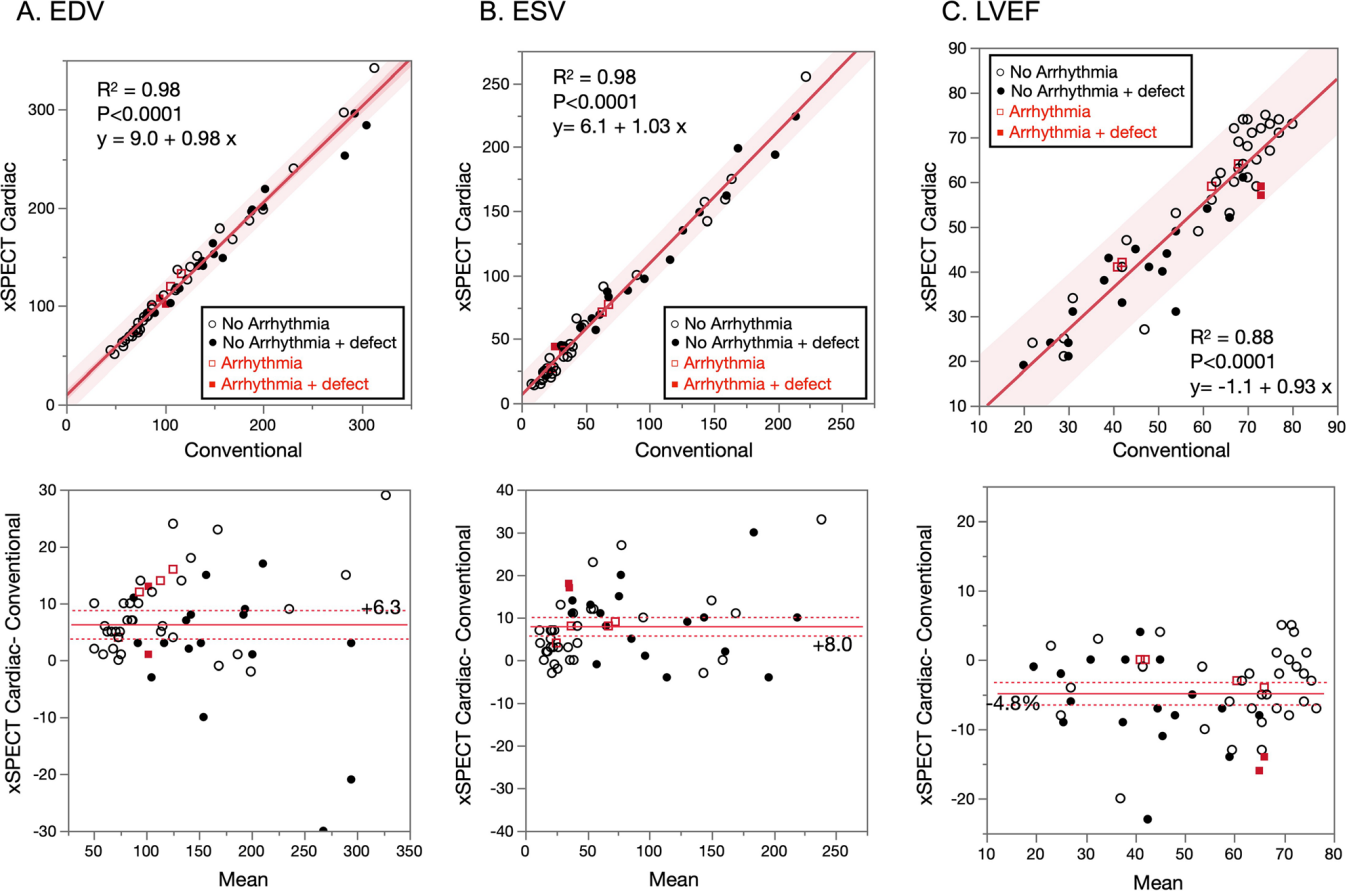
Comparison of left ventricular (LV) function for end-diastolic volume (EDV), end-systolic volume (ESV), and ejection fraction (EF). Upper panels: linear regression lines and confidence shaded fits for individual values (red). Lower panels: Red and dashed lines indicate mean differences and upper and lower 95% levels of the mean, respectively. The differences between xSC and conventional Flash3D are +6.3 mL (lower -upper 95% range, 3.8 - 8.8 mL), +8.0 mL (5.8 - 10.2 mL), and -4.8% (-6.4% - -3.2%), for EDV, ESV, and EF, respectively. Red solid and open squares, arrhythmic beats ≥ 20% with and without myocardial infarction, respectively. Black solid and open circles, no significant arrhythmic beats with and without myocardial infarction, respectively

Correlations between xSC and conventional methods were computed for EDV (R^2^ = 0.98, p < 0.0001), ESV (R^2^ = 0.98, p < 0.0001), and LVEF (R^2^ = 0.88, p < 0.0001) (Figure 6). The EDV and ESV were respectively higher by 6 and 8 mL, and LVEF was 5% lower for xSC compared with conventional SPECT methods. Correlations between these variables and echocardiographic values did not significantly differ between the xSC and conventional methods (R^2^ = 0.59 and 0.58 for EDV, 0.78 and 0.78 for ESV and 0.82 and 0.78 for LVEF, respectively). In the group with near-normal perfusion and function (n = 24, M: F, 18: 6), mean EDVs, ESVs and LVEFs, for males and females were 91 (10 - 90% range, 62-141) and 91 (55-118) mL; 33 (18-62) mL and 27 (15-44), mL and 65% (56%-74%) and 70% (62%-74%), respectively, using the xSC method.

### Effects of arrhythmia: prospective vs. retrospective gating methods

The prospective image generation using list-mode and clinical prospective acquisitions allowed comparisons of two gating strategies with separately acquired data. We also confirmed that the beat histogram characteristics remained unchanged in a back-to-back study as described.^19^

**Figure 7:**
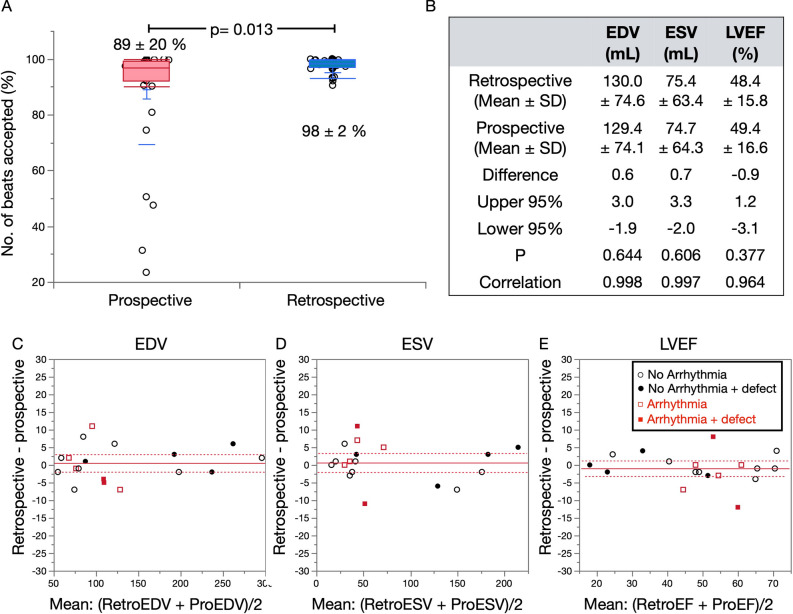
Comparison of prospective gates with 20% window and retrospective gates. (A) Number of accepted beats, (B) end-diastolic volume (EDV), end-systolic volume (ESV), and ejection fraction (EF). Brand-Altman plots for (C) EDV, (D) ESV, and (E). The red solid and dashed lines are the mean difference and 95% confidence limits of the mean, the same as indicated in Figure 6

**Figure 8:**
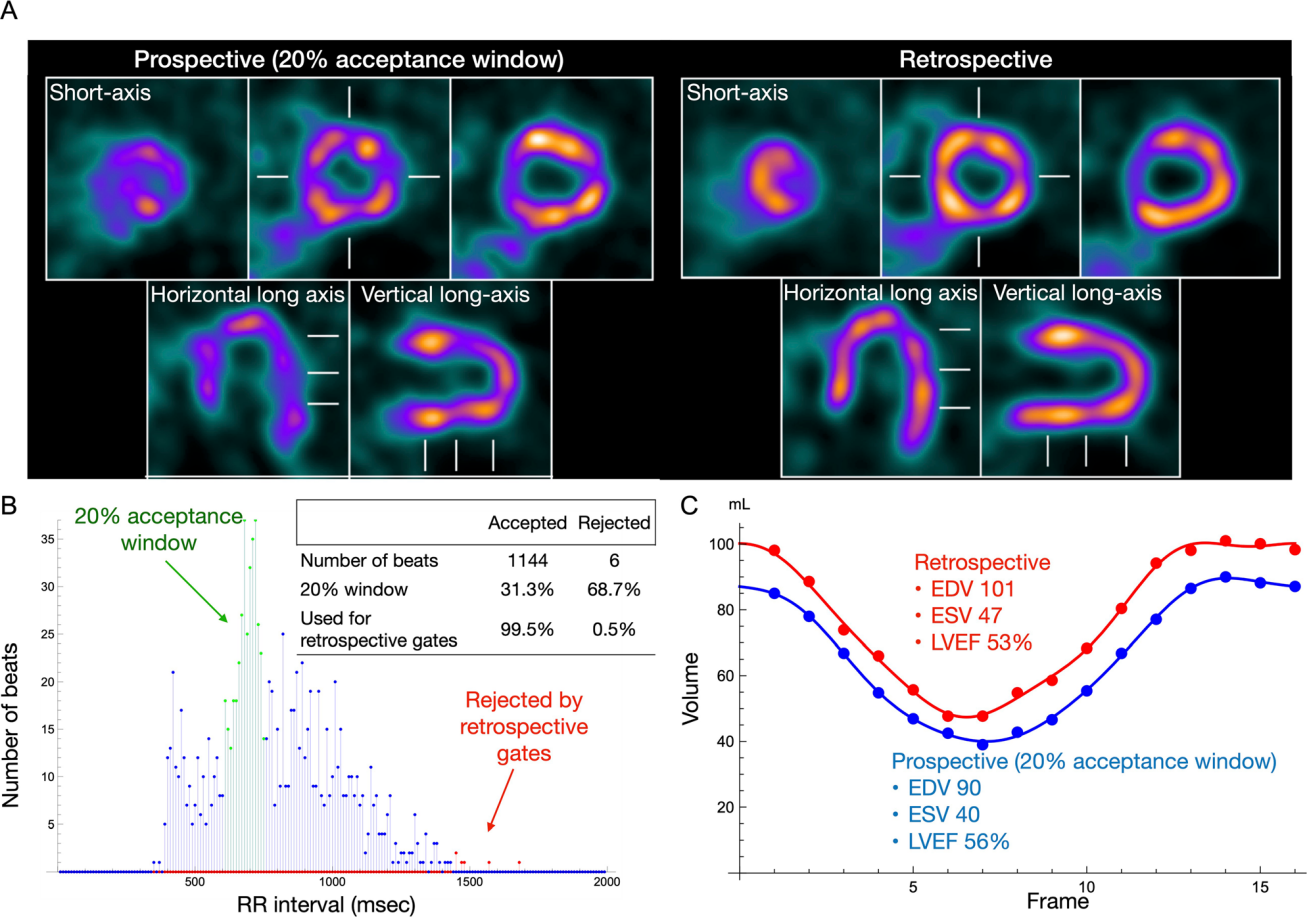
Conventional prospective gating (20% window allowance) and retrospective gating images created by list-mode data. Ordered subset conjugate gradient method was used for this figure. (A) Noise levels are affected by numbers of accepted beats (31.3% *vs*. 99.5% for prospective and retrospective gating, respectively). (B) Beat length histogram during acquisition shows distribution of acquired and rejected beats. (C) Volume curves and functional parameters

The following results were generated from a comparison of retrospective and prospective gating methods, in which all corrections were applied. When all heart-beat lengths were analyzed, conventional prospective gating and xSPECT with a 20% window accepted 89% ± 20% and 98% ± 2% of beats, respectively (Figure 7A). A significant portion (≥ 20%) of beats in six patients was rejected. Prospective gating by list-mode data derived from a selected patient group resulted in no significant differences between the two methods with respect to EDV, ESV, and LVEF (Figure 7B). However, total counts used for reconstruction were increased by the retrospective method, resulting in less noise and, thus, better image quality (Figure 8). In an example shown in this figure, retrospective gating used 99.5% of the data with only 0.5% outlier values, while prospective gating with a 20% window used only 31.3% of the data.

## Discussion

The ideal characteristics of SPECT images include accurate measurements of tracer accumulation and quantitative images that are sufficient for evaluating regional myocardial uptake and estimating functional parameters. Attenuation and scatter correction incorporated into an ordered subset/maximum-likelihood expectation maximization (OSEM/MLEM) method has been recommended to achieve optimal SPECT image reconstruction.^20–22^ Patient motion correction during data acquisition, if present, also contributes to accurate quantitation.^23^ Electrocardiogram gating to exclude arrhythmic heartbeats is essential when imaging a beating heart and further correction of respiratory motion might improve image quality and patient-by-patient variability.^24–26^ However, the combined effects of such corrections have yet to be determined and they are not necessarily applied in clinical practice. We found that the integrated corrections for the SPECT images using a list-mode data acquisition are technologically and clinically feasible and significantly improved image quality by visual assessment without any noticeable pitfalls. We also assessed LV function by retrospective gating while fully maintaining the acquisition counts during data acquisition.

### Attenuation and scatter correction

Attenuation and scatter corrections are considered indispensable to ensure quantitative accuracy. We found that the myocardial wall count distribution became homogeneous in the polar map analysis (Figure 2). Although the apical segment showed lower counts in the attenuation corrected images, such anatomical apical thinning was also confirmed by X-ray CT.^27^ Attenuation correction has been established since the mid 1990s as a strategy to improve the diagnostic accuracy of the inferior wall.^20^ The American Society of Nuclear Cardiology (ASNC) and the Society of Nuclear Medicine also affirmed that AC should be regarded as an evolving standard for SPECT.^28^ However, users found the method cumbersome in clinical practice, as well as hardware and manufacturer dependent.^29^ With advances in SPECT-CT technology, the ASNC has recommended the standard application of AC^21^ in nuclear medicine facilities. To avoid misinterpretation by AC artifact, a combination of conventional non-AC image and adjunct AC images are generally recommended. Scatter correction is also required to compensate for over-correction in attenuated deep regions. However, only 30% of Japanese institutions use both AC and SC. In the present study, activity in inferior walls was mistakenly identified as reduced in as many as 23% of non-AC cases, while application of AC and SC allowed correct identification of these cases as normal. Figure 1A shows that the small perfusion defects could be more appropriately evaluated by appropriate corrections.

### Inter-view correction for tomographic inconsistency during acquisition

Another improvement applied herein was the mitigation of tomographic inconsistencies incurred during projection image acquisition such as from motion. We quantified inconsistencies by determining shift vectors in the iterative reconstruction process including AC and SC, as a proxy for motion. The xSC method used a fully automated method during reconstruction that allowed rigid axial and transaxial shifts to improve tomographic consistency as assessed by the reconstruction method. Fully 4D motion correction was not possible given the multi-channel collimator image formation resulting in noisy data. Practical approximations must therefore be made to avoid having too many variables for too little data, and thus we corrected only in axial and transaxial direction and for each view separately. Therefore, it was not an anatomically correct motion correction, but a mitigation, as only tomographic consistency within the rigid translational model can be improved, regardless of the actual root cause of the inconsistency. We demonstrated the effect of organ motion on image quality, by applying patient-derived shift vectors on a resting phantom to visualize the effect (Figures 4-5), emulating the effect on image quality given the presence of the inconsistency determined by the *inter-view* motion correction. We showed the potential of the methods to improve count distribution pattern and edge. However, the impact of the *inter-view* motion correction in clinical images was not apparent. For one the histogram only showed very small shifts of only a few mm, indicating essentially no relevant motion as assessed by the rigid-body translational model. Nonetheless, the method was safely applied during reconstruction without generating harmful artifacts and contributed to steadying the procedure.

### Intra-view motion corrections

In principle, respiratory motion correction should also contribute to more accurate quantitation and to improve patient-by-patient variability, but its role has not yet been established in clinical practice. External devices have been used for detecting respiratory motions. An elastic strap placed on the torso of patients is used to generate respiratory bins during SPECT examinations.^25^ Anzai pressure belt (Anzai Medical, Tokyo, Japan) was able to track displacement of the abdominal wall, and applied to dynamic positron-emission tomography imaging.^30^ In contrast, data-driven methods for extracting respiratory surrogate signals from SPECT list-mode data have been proposed, and a validation study of 67 patients found a mean correlation against the Anzai belt of 0.81 ± 0.17.^16^ We used the data-driven approach to the present study as it is convenient for clinical practice. In general, respiratory motion patterns vary among patients and cannot be estimated in advance as indicated by the boxplot of each respiratory gate (Figure 4). Regarding the effect of respiratory gating on LV functional assessment, a recent study found that dual respiratory and cardiac gating slightly increased the average LV volume from 77.1 to 79.8 mL compared with cardiac gating.^31^ Our results using respiratory gating agreed with those of the slightly higher volume in that study and were considered reasonable because the degree of blurring due to breathing motion was small but non-negligible. In particular when twofold respiratory motion was assumed (range of motion 12 mm), which could happen in clinical studies, the effects were apparently visible on the still phantom image (Figure 5).

### List-mode-based retrospective gating

List-mode acquisition and the retrospective cardiac gating as a standard procedure is also a characteristic of this study. The results with retrospective gating closely correlated with conventional clinical frame-mode acquisition with a fixed window (for example, at 20%) and the auto-tracked RR interval. Moreover, correlations of functional parameters of EDV, ESV, and LVEF were acceptable even when prospective gating data with a 20% window were simulated using the acquired list-mode data. Data acquisition with variable beat length in patients with arrhythmia resulted in reduced total counts especially during the last part of RR intervals, which subsequently increased noise in the reconstructed images. Retrospective gating from list-mode used 98% of the data and contributed to stable image reconstruction. Since list-mode data included all information about detected gamma-ray position, energy, and timing of events, the most important advantage is the generation of images of almost equal statistical quality under arbitrary beat histogram conditions. Although such quantitative merits are established and applied to clinical gated blood-pool investigations and animal studies,^32–34^ their application to gated SPECT studies has not become standard. This is partly due to the amount of time required for reconstruction and cumbersome steps compared to the conventional frame-mode acquisition. However, it seems to be an acceptable option considering versatile processing applications for patients with significant arrhythmia. The processing time for retrospective gating including all corrections was <5 minutes as a research program, which can be shortened to ~1 min with a dedicated processing system available at present.

The acceptable fraction of beats must be considered when imaging patients with serious arrhythmia and large variations in RR intervals. Frequent premature contractions and subsequent beats in patients have commonly been excluded. In contrast, LVEF differs in patients with atrial fibrillation with and without windowing, and how it varies with cycle has been determined in a study that included a gated blood pool.^35^ A comparison of averages between narrow-and non-windowed LVEF revealed a good correlation between the two approaches. Therefore, the LVEF averaged by including most of the beats might be used as a standard even for gated SPECT if LVEF is the purpose of the study. However, contamination with arrhythmic beats could interfere measurement accuracy if the purpose of the study is to determine functional diastolic filling.^32^ A study of the influence of various arrhythmia types on gated SPECT images revealed minimal changes in LVEF (1% ± 4%) and volumes (2% ± 9%) compared to controls, whereas end-frame count loss rendered wall thickening calculations and perfusion analyses inaccurate.^36,37^

### Integration of all corrections

The question arises as to whether integrating all corrections is feasible, effective, and practical. Although acquisition and reconstruction conditions between the conventional and xSC methods differed significantly, our aim of this study was not just to compare clinical conditions and highly integrated research condition but also to apply the new corrections to clinical practice. Myocardial SPECT images include fundamental flaws essentially derived from reconstruction by multiple projection images, attenuation, scatter, patient motion, cardiac motion, respiratory movement, and the possible inclusion of arrhythmia. Moreover, the effects of these confounding factors depend on patients and their conditions during data acquisition. For example, AC, motion, and arrhythmia correction could be applied to patients with obesity, unexpected motion, and frequent aberrant beats, respectively. However, we reduced patient-to-patient variability by correcting all known factors that might cause artifacts. Important aspects are new possible artifacts created by advanced correction methods as those associated with AC during the early 2000s.^38^ It is reported that when combined with correction for motion, depth-dependent blurring, and attenuation correction significantly improved diagnostic accuracy compared to the motion correction alone.^22^ Notably, integrating all corrections did not generate any harmful artifacts in this study. Lastly, although improving quantitative values by integrating all possible correction methods is desirable, whether pursuing true quantitation would definitively improve clinical diagnostic ability requires further investigation.^26^

### Limitations

One limitation of this study was that we applied corrections only to rest studies. Since this study aimed to determine the technical feasibility of integrated corrections, we examined imaging characteristics and functional aspects of patients with myocardial infarction.

The ground truth for a clinical diagnosis of myocardial infarction relied on patient history, ECG, echocardiography, and coronary angiography findings. Information about coronary flow or fractional flow reserve was not included. The next steps will be to validate the effectiveness and diagnostic utility of the method in stress-rest studies. Our conventional method did not include CT AC. Images with AC are still not popular in Japan, which is partly due to a mean BMI of 23 (only two of our patients had a BMI > 30) and an acquisition arc of 360°. Under such conditions, a beneficial effect of AC has not been recognized in clinical practice. Moreover, the contributions of corrected attenuation, scatter, motion, tomographic inconsistency, and gating methods were not separately and independently evaluated, because an integrated corrections were applied to avoid numerous permutations.

## Conclusions

SPECT images with attenuation and scattering correction, *inter-view* correction of tomographic inconsistency, *intra-view* motion correction from data-driven respiratory gating, and list-mode-based retrospective ECG gating improved resolution and could deliver repeatable quantitative results. The diagnostic accuracy of perfusion defects using xSPECT Cardiac is at least equal to using conventional SPECT imaging.

## New knowledge gained

Myocardial SPECT images yield better image quality when attenuation, scattering, respiratory movement, tomographic inconsistency, arrhythmia, and list-mode-based retrospective gating were corrected. Integrated corrections are technologically feasible, creating no harm, and could be effective for clinical studies.

### Supplementary Information

Below is the link to the electronic supplementary material.

Supplementary file1 (DOCX 25 KB)

Supplementary file2 (PPTX 9266 KB)

Supplementary file3 (DOCX 144 KB)

## Abbreviations

AC: Attenuation correction
EDV: End-diastolic volume
EF: Ejection fraction
ESV: End-systolic volume
FBP: Filtered back projection
ITC: Improving tomographic consistency
OSEM: Ordered-subset expectation maximization
SC: Scatter correction
SPECT: Single-photon emission-computed tomography
xSC: XSPECT Cardiac (prototype reconstruction method)

## Footnotes

1 The prototype xSPECT Cardiac (xSC) using Symbia Intevo data is essentially equivalent to a feature of the Symbia Pro.specta™ (Siemens Healthineers, Erlangen, Germany) that is now on the market. Symbia Pro.specta might not be released in all regions and countries.

2 Prototype of the commercial product "MC.pro" which is further explained in the supplementary material

3 prototype of the commercial product "rMC.pro" which is further explained in the supplementary material.

4 We differentiate between center-of-light and center-of-mass. The latter is estimated best by an anatomical imaging modality, the former, however, is the effective distribution of emission integrated over the dwell time, which can be longer than the duration of the motion itself.
