# Supplementary material for: Myocardial perfusion imaging with retrospective gating and integrated correction of attenuation, scatter, respiration, motion, and arrhythmia
Source: J Nucl Cardiol. 2023 Sep 27;30(6):2773–89. doi: 10.1007/s12350-023-03374-5 (PMC10682219; doi:10.1007/s12350-023-03374-5)
Supplement: Supplementary file 1 — Supplementary file1 (DOCX 25 KB) [file 12350_2023_3374_MOESM1_ESM.docx]

Supplement for

**Myocardial perfusion imaging with retrospective gating and integrated correction of attenuation, scatter, respiration, motion, and arrhythmia**

Journal of Nuclear Cardiology 2023

Kenichi Nakajima ^1^, Takayuki Shibutani ^2^, Francesc Massanes ^3^, Takeshi Shimizu ^3^, Shohei Yoshida ^4^, Masahisa Onoguchi ^2^, Seigo Kinuya ^5^, A. Hans Vija ^3^

1. Functional Imaging and Artificial Intelligence, Kanazawa University, Kanazawa, Japan, E mail: nakajima@med.kanazawa-u.ac.jp

2. Quantum Medical Technology, Institute of Medical, Pharmaceutical and Health Sciences, Kanazawa University, Kanazawa, Japan

3. Siemens Medical Solutions USA, Inc. Molecular Imaging, Hoffman Estates, IL, USA

4. Department of Cardiovascular Medicine, Kanazawa University Graduate School of Medical Science, Kanazawa, Kanazawa, Japan

5. Department of Nuclear Medicine, Kanazawa University, Kanazawa, Japan

*Inter-view* and *intra-view* motion corrections for SPECT image reconstruction

**A. *Inter-view* motion correction** allows to reduce tomographic inconsistencies and potentially mitigates a build-up of image artifacts as updates progress, and was designed to be compatible with iterative reconstruction, yet is limited by poor count statistics and the fundamental limitations of the image formation.

We first describe the current method to highlight the commonality and the key difference of this new approach. The current commercially available proprietary semi-automatic method is based on cross-correlation to detect motion^1, 2^. It estimates axial and transaxial motion in each projection view to mitigate cardiac motion induced by patient motion or cardiac creep and in use since 2001. A user defined box is placed to encompass the projection of the myocardium in the starting view allows for the tracking of the cardiac projection using cross-correlation maximization, where the position of the maximum determines the horizontal heart center. The amplitude of motion is determined by sine wave fitting. Transaxial motion is estimated based on integral sinograms, while vertical motion estimation is based on linograms. The user must carefully choose the sino and/or linogram ranges to ensure a stable and reasonable shift of the projection views. The projections are shifted view-by-view in axial and transaxial direction, resulting in the motion corrected projection data, prior to reconstruction. In addition, the user can choose to override the shifts by manually shifting individual views. The impact of this correction method is assessed by visual inspection of the resulting image, often leading to a trial-and-error approach repeating correction and reconstruction. Fundamentally, this method also suffers, from lack of attenuation correction, where contrast can fall so low that edge detection and subsequent fits become unreliable, and potentially inducing erroneous shifts. As this method is applied before reconstruction and is not connected to any optimization metric within iterative reconstruction, erroneous shifts can be made which can worsen artifacts with increasing iteration leading to a time-consuming trial-and-error approach.

The xSPECT Cardiac (xSC; prototype) method used in this study overcomes these issues using a fully automated method applicable to all study types including cardiac study, which allows for rigid axial and transaxial shifts that are measured within and designed for the iterative reconstruction process. The shift vectors are estimated in a preceding dedicated Improving Tomographic Consistency (ITC)-reconstruction with the same attenuation correction (AC)^[[1]](#footnote-1)^ and scatter correction (SC) as the target xSC reconstruction, however, at reduced updates and allowing for computation of shift vectors as a result of two-dimensional rigid body mutual-information based registration between data model and data. The xSC reconstruction restarts using the shift vectors from the preceding ITC reconstruction by updating the 3D rotation matrix, thereby improving tomographic consistency. All reconstructed images were displayed as transaxial images and subsequently analyzed. As of 2022, this feature has been productized as MC.pro^TM^.

**B. *Intra-view* motion correction** refers to mitigating for motion during the dwell at a specific viewing angle. While we above addressed the tomographic inconsistency between views we now address the impact of motion during the dwell time, for both continuous or discontinuous rotation. Respiration is typically the main cause for such motion, and inconsistencies may also appear over multiple views. In particular, if the dwell time is roughly the same or shorter than the breathing period motion impacts both intra- and inter-view projection. If the motion period is much faster than dwell time the intra-view motion results in “smearing” of the projected object, and it may even be identical in each view in which case ITC would not find any inconsistency. However, that needs to be addressed, and we thus introduced a method to mitigate for such shape deformation in the projection view. We only correct for axial motion, and while it is not able to fully mitigate for the true three-dimensional respiratory motion it is a doable correction given the count statistics and image formation. Unlike in the *inter-view* method, this correction occurs prior to framing of the data, and thus must occur prior to ITC. We use data-driven dimensionality reduction with a non-linear Laplacian Eigenmap approach based on the assumption that the data lay on a lower-dimensional manifold; namely a temporal sequence of 200 ms micro-frames to estimate six respiratory gates for each view. By subdividing the projection view within a dwell time (here: 15 seconds) into six estimated respiratory gates one has a somewhat better statistics to enable an estimate of the center-of-light^[[2]](#footnote-2)^ motion in that view and thus subsequently perform an intra-view correction of respiration-induced axial motion. The center-of-light for respiratory-gated views (RGV) within the projection view could be measured and then be shifted to a common average location, prior to summation into the viewing frame. This approach mitigated respiration-induced blurring and shape deformation of the VOI in projection view after summing the mean shifted RGVs and creating a framed dataset. As of 2022, this feature has been productized as rMC.pro^TM^.

References

1. Eisner RL, Noever T, Nowak D, Carlson W, Dunn D, Oates J et al. Use of Cross-Correlation Function to Detect Patient Motion during SPECT Imaging. Journal of Nuclear Medicine 1987;28:97-101.

2. Wei G-Q, Qian J, Chen EQ, Engdahl JC. Variable-length correlation method for motion correction in SPECT myocardial perfusion imaging. United States; 2002, Patent Number: 6473636

1. If CT AC is not available, the software deploys dedicated emission data-driven AC to avoid erroneous shift vector computation. [↑](#footnote-ref-1)
2. We do differentiate between center-of-light and center-of-mass, which do not need to be the same in multi modal imaging. The latter, often obtained from a CT is typically a snap shot of the object, while the former may be a “smear”. [↑](#footnote-ref-2)
