# Supplementary material for: Myocardial perfusion imaging with retrospective gating and integrated correction of attenuation, scatter, respiration, motion, and arrhythmia
Source: J Nucl Cardiol. 2023 Sep 27;30(6):2773–89. doi: 10.1007/s12350-023-03374-5 (PMC10682219; doi:10.1007/s12350-023-03374-5)
Supplement: Supplementary file 2 — Supplementary file2 (PPTX 9266 KB) [file 12350_2023_3374_MOESM2_ESM.pptx]

## Slide 1
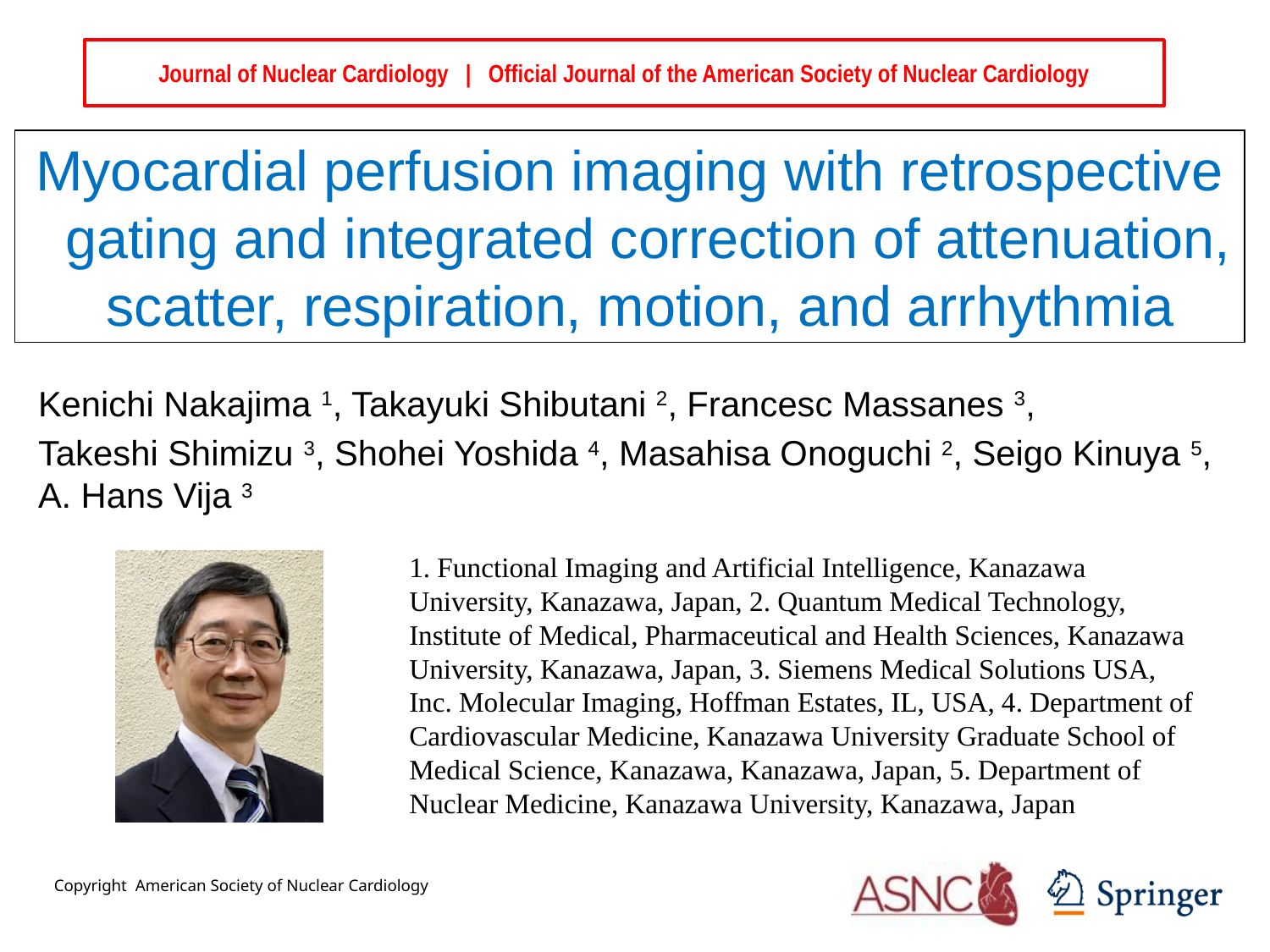

Journal of Nuclear Cardiology | Official Journal of the American Society of Nuclear Cardiology
# Myocardial perfusion imaging with retrospective gating and integrated correction of attenuation, scatter, respiration, motion, and arrhythmia
Kenichi Nakajima 1, Takayuki Shibutani 2, Francesc Massanes 3,
Takeshi Shimizu 3, Shohei Yoshida 4, Masahisa Onoguchi 2, Seigo Kinuya 5, A. Hans Vija 3
1. Functional Imaging and Artificial Intelligence, Kanazawa University, Kanazawa, Japan, 2. Quantum Medical Technology, Institute of Medical, Pharmaceutical and Health Sciences, Kanazawa University, Kanazawa, Japan, 3. Siemens Medical Solutions USA, Inc. Molecular Imaging, Hoffman Estates, IL, USA, 4. Department of Cardiovascular Medicine, Kanazawa University Graduate School of Medical Science, Kanazawa, Kanazawa, Japan, 5. Department of Nuclear Medicine, Kanazawa University, Kanazawa, Japan
Copyright American Society of Nuclear Cardiology

## Slide 2
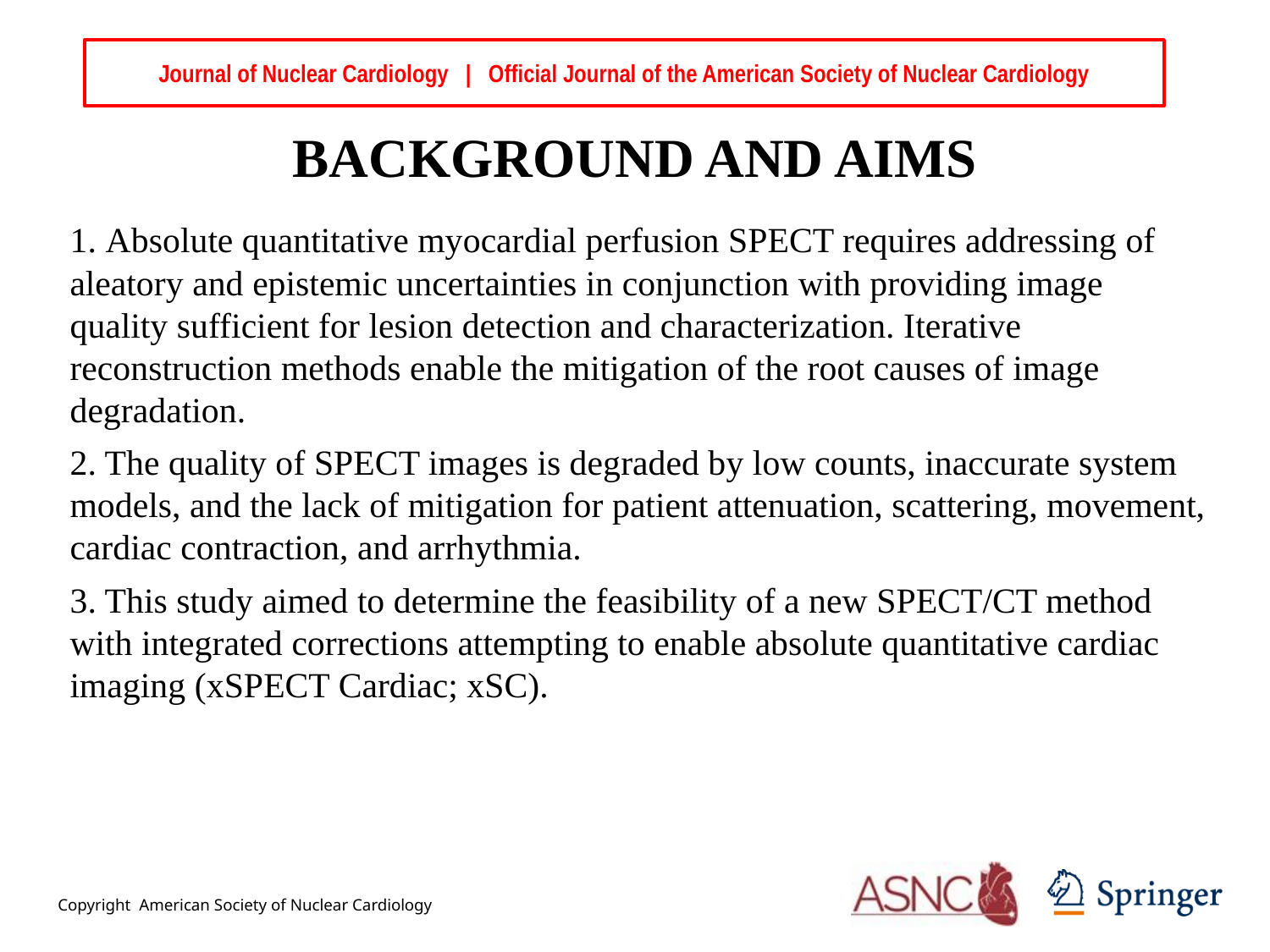

Journal of Nuclear Cardiology | Official Journal of the American Society of Nuclear Cardiology
# BACKGROUND AND AIMS
1. Absolute quantitative myocardial perfusion SPECT requires addressing of aleatory and epistemic uncertainties in conjunction with providing image quality sufficient for lesion detection and characterization. Iterative reconstruction methods enable the mitigation of the root causes of image degradation.
2. The quality of SPECT images is degraded by low counts, inaccurate system models, and the lack of mitigation for patient attenuation, scattering, movement, cardiac contraction, and arrhythmia.
3. This study aimed to determine the feasibility of a new SPECT/CT method with integrated corrections attempting to enable absolute quantitative cardiac imaging (xSPECT Cardiac; xSC).
Copyright American Society of Nuclear Cardiology

## Slide 3
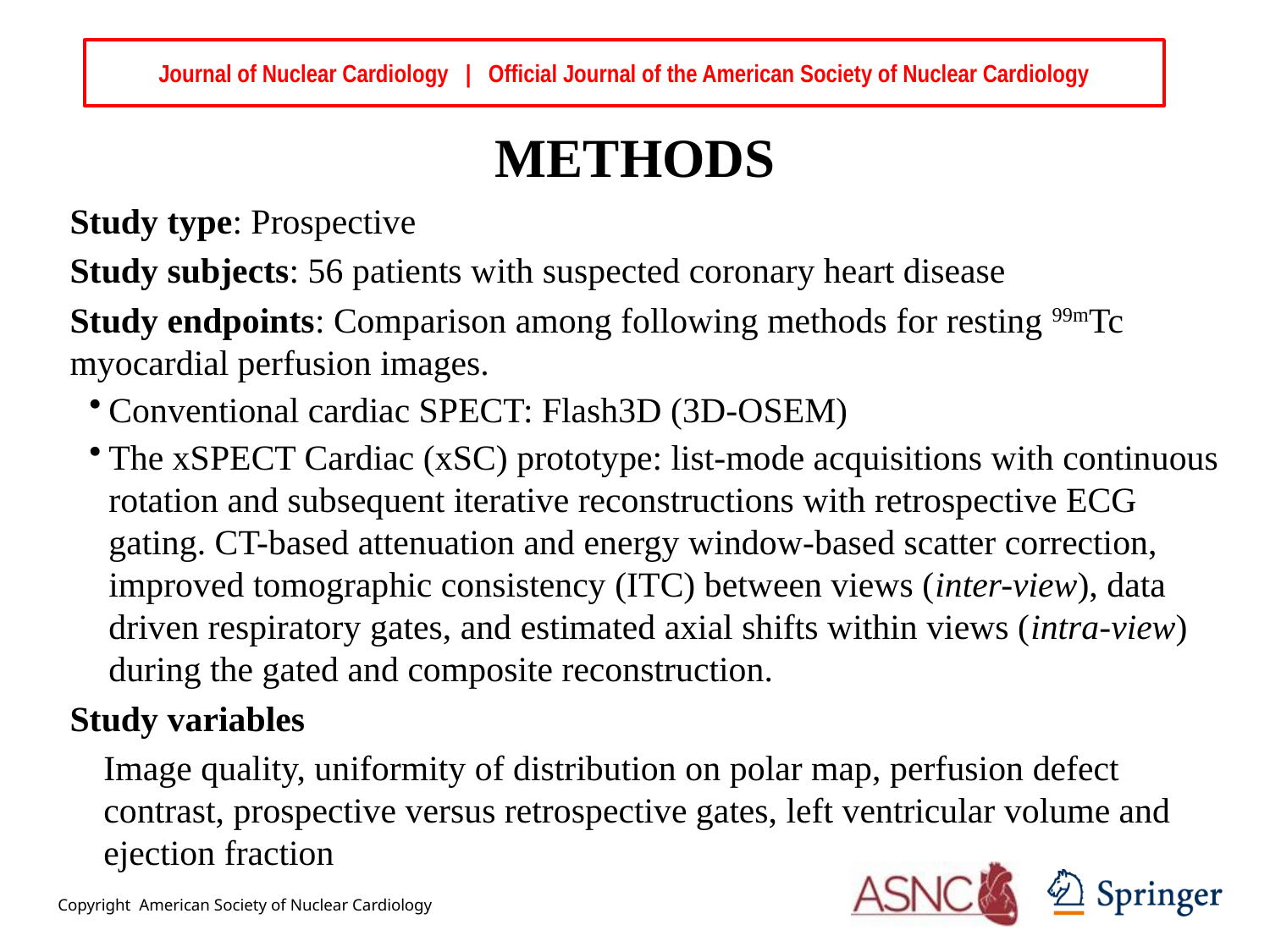

Journal of Nuclear Cardiology | Official Journal of the American Society of Nuclear Cardiology
# METHODS
Study type: Prospective
Study subjects: 56 patients with suspected coronary heart disease
Study endpoints: Comparison among following methods for resting 99mTc myocardial perfusion images.
Conventional cardiac SPECT: Flash3D (3D-OSEM)
The xSPECT Cardiac (xSC) prototype: list-mode acquisitions with continuous rotation and subsequent iterative reconstructions with retrospective ECG gating. CT-based attenuation and energy window-based scatter correction, improved tomographic consistency (ITC) between views (inter-view), data driven respiratory gates, and estimated axial shifts within views (intra-view) during the gated and composite reconstruction.
Study variables
Image quality, uniformity of distribution on polar map, perfusion defect contrast, prospective versus retrospective gates, left ventricular volume and ejection fraction
Copyright American Society of Nuclear Cardiology

## Slide 4
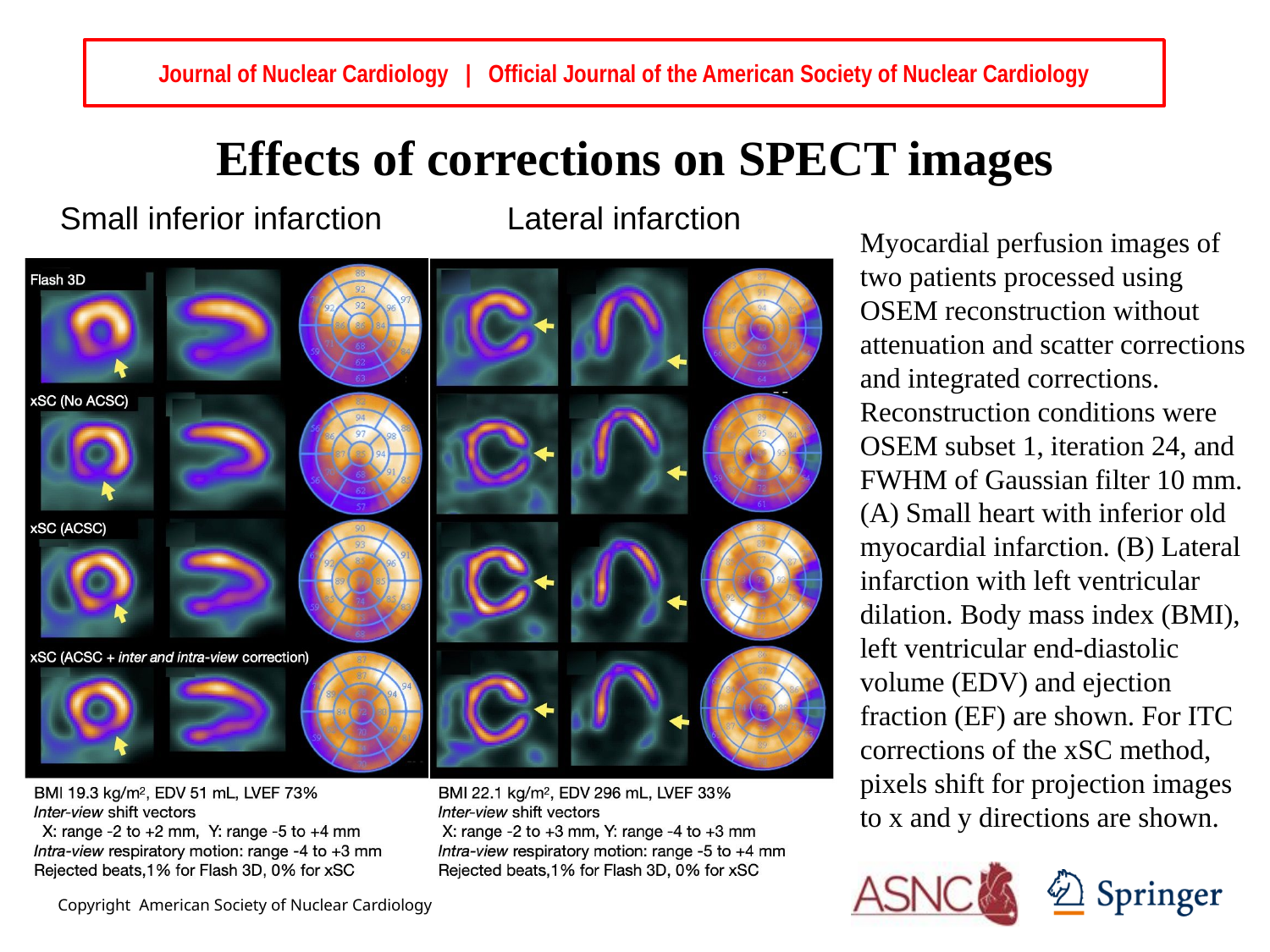

Journal of Nuclear Cardiology | Official Journal of the American Society of Nuclear Cardiology
# Effects of corrections on SPECT images
Small inferior infarction
Lateral infarction
Myocardial perfusion images of two patients processed using OSEM reconstruction without attenuation and scatter corrections and integrated corrections.
Reconstruction conditions were OSEM subset 1, iteration 24, and FWHM of Gaussian filter 10 mm.
(A) Small heart with inferior old myocardial infarction. (B) Lateral infarction with left ventricular dilation. Body mass index (BMI), left ventricular end-diastolic volume (EDV) and ejection fraction (EF) are shown. For ITC corrections of the xSC method, pixels shift for projection images to x and y directions are shown.
Copyright American Society of Nuclear Cardiology

## Slide 5
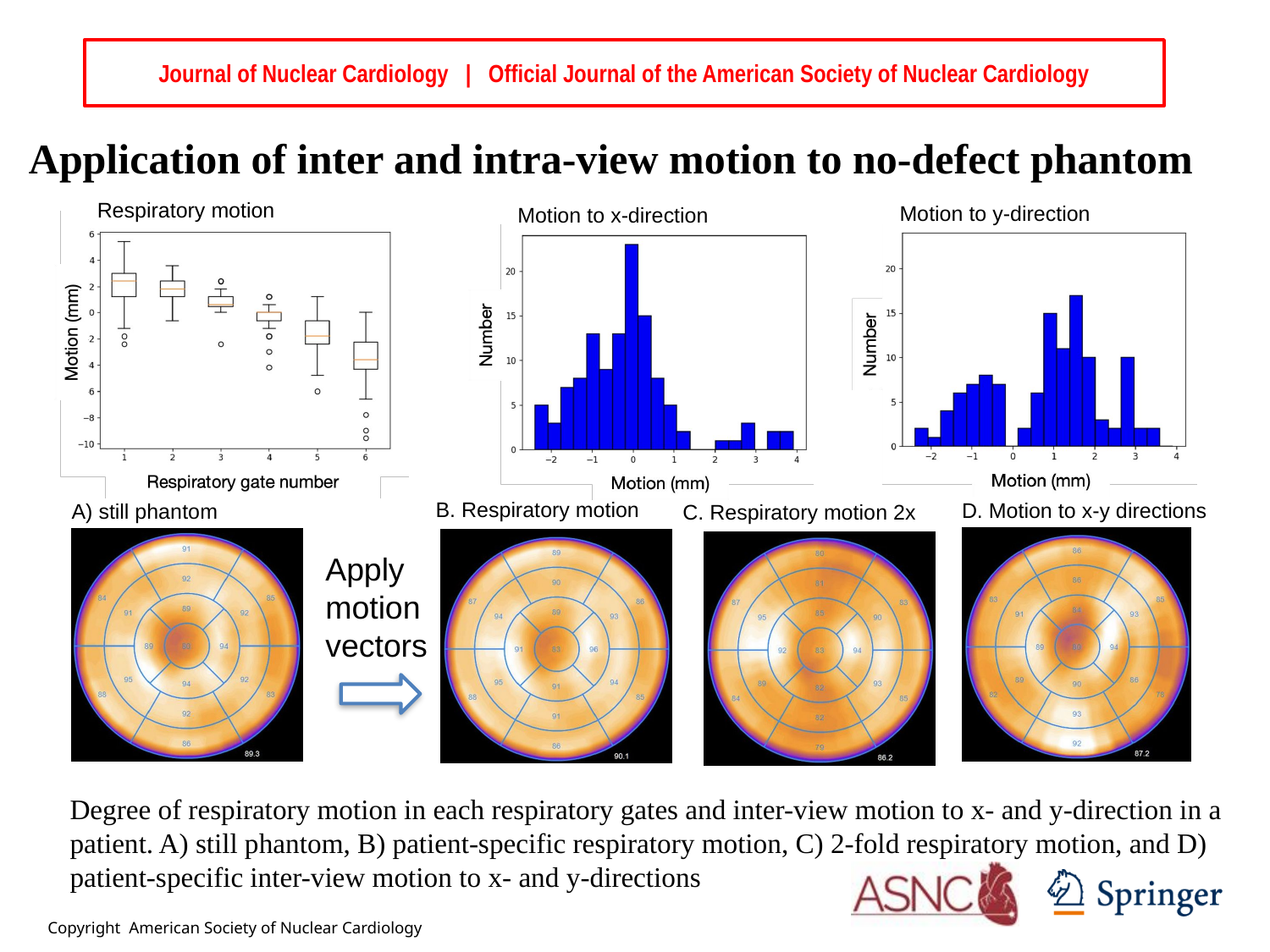

Journal of Nuclear Cardiology | Official Journal of the American Society of Nuclear Cardiology
# Application of inter and intra-view motion to no-defect phantom
Respiratory motion
Motion to y-direction
Motion to x-direction
B. Respiratory motion
D. Motion to x-y directions
A) still phantom
C. Respiratory motion 2x
Apply motion vectors
Degree of respiratory motion in each respiratory gates and inter-view motion to x- and y-direction in a patient. A) still phantom, B) patient-specific respiratory motion, C) 2-fold respiratory motion, and D) patient-specific inter-view motion to x- and y-directions
Copyright American Society of Nuclear Cardiology

## Slide 6
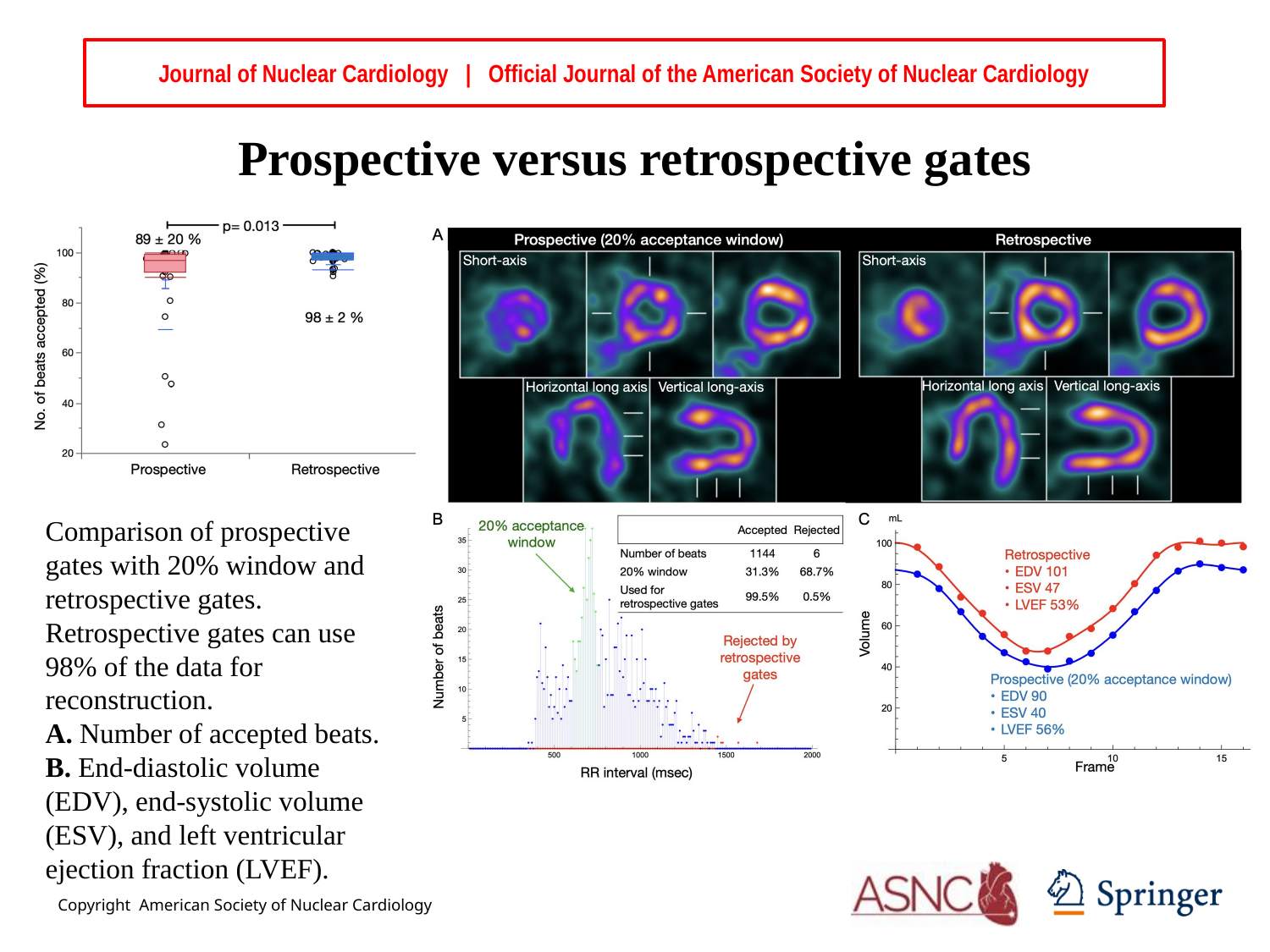

Journal of Nuclear Cardiology | Official Journal of the American Society of Nuclear Cardiology
# Prospective versus retrospective gates
Comparison of prospective gates with 20% window and retrospective gates. Retrospective gates can use 98% of the data for reconstruction.
A. Number of accepted beats. B. End-diastolic volume (EDV), end-systolic volume (ESV), and left ventricular ejection fraction (LVEF).
Copyright American Society of Nuclear Cardiology

## Slide 7
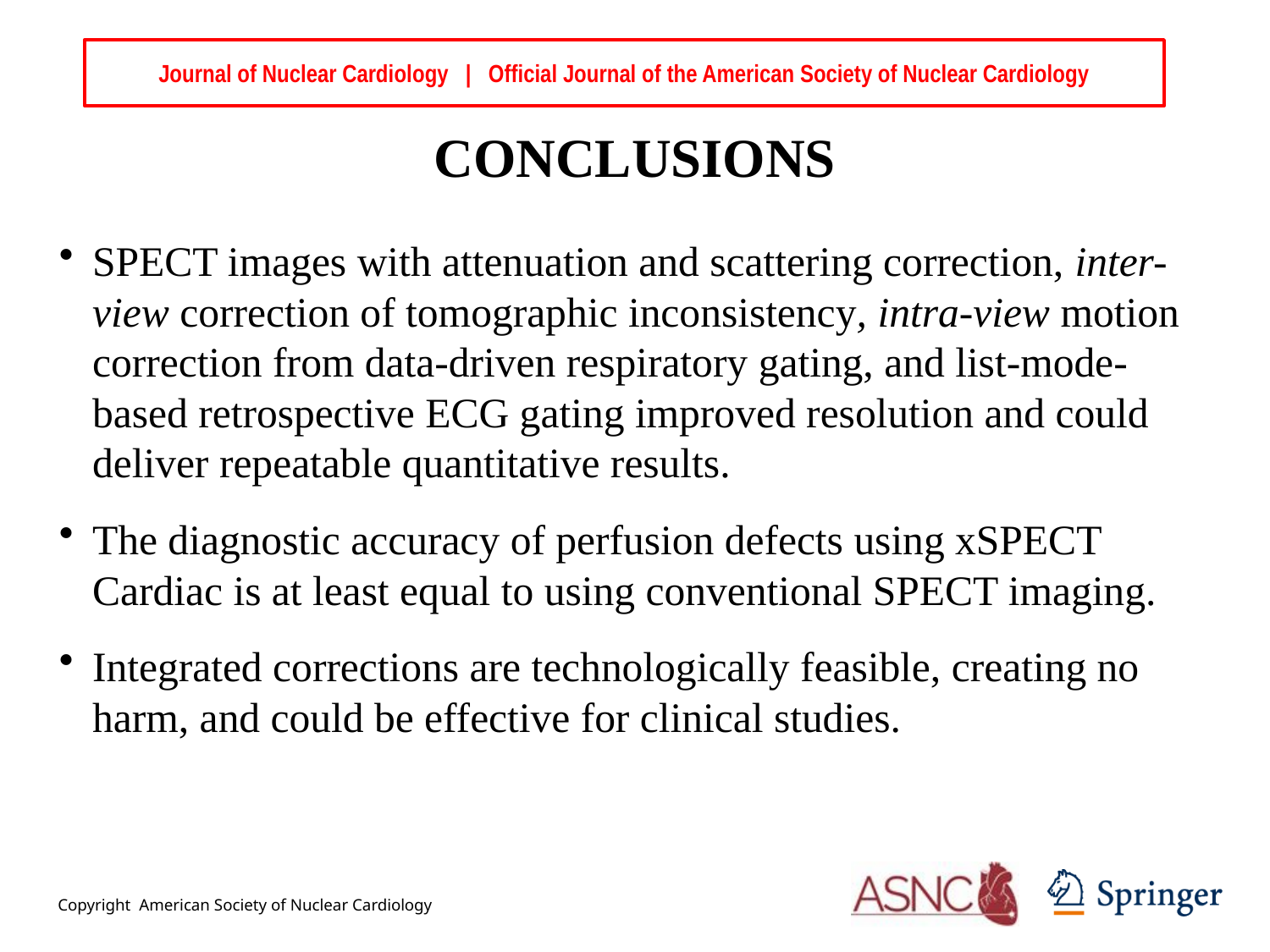

Journal of Nuclear Cardiology | Official Journal of the American Society of Nuclear Cardiology
# CONCLUSIONS
SPECT images with attenuation and scattering correction, inter-view correction of tomographic inconsistency, intra-view motion correction from data-driven respiratory gating, and list-mode-based retrospective ECG gating improved resolution and could deliver repeatable quantitative results.
The diagnostic accuracy of perfusion defects using xSPECT Cardiac is at least equal to using conventional SPECT imaging.
Integrated corrections are technologically feasible, creating no harm, and could be effective for clinical studies.
Copyright American Society of Nuclear Cardiology
